# Evaluation of Salivary Biomarkers and Spirometry for Diagnosing COPD in Non-Smokers and Smokers of Polish Origin

**DOI:** 10.3390/biomedicines12061206

**Published:** 2024-05-29

**Authors:** Magdalena Rudzinska-Radecka, Bartłomiej Bańcerowski, Robert Marczyński, Debjita Mukherjee, Tomasz Sikora, Karolina Morawska, Agnieszka Mielczarek, Marcin Moździerski, Bogdan Hajduk, Beata Kotowicz

**Affiliations:** 1 Recumed Ltd., 05-092 Łomianki, Poland; 2 Institute of Physical Chemistry, Polish Academy of Sciences, 01-224 Warszawa, Poland; 3 Military Institute of Chemistry and Radiometry, 00-910 Warsaw, Polandkarmorawska@gmail.com (K.M.); 4 Department of Conservative Dentistry and Endodontics, Medical University of Warsaw, 02-097 Warsaw, Poland; agnieszka.mielczarek@wum.edu.pl; 5 TS Out-Patients Clinic for Cardiovascular and Pulmonary Diseases, 01-460 Warsaw, Poland; b.hajduk1@vp.pl; 6 Cancer Biomarker and Cytokines Laboratory Unit, Maria Skłodowska-Curie National Research Institute of Oncology, 02-781 Warsaw, Poland; beata.kotowicz@nio.gov.pl

**Keywords:** salivary biomarkers, COPD, C-reactive protein, procalcitonin, neutrophil elastase, alpha-1 antitrypsin, Polish origin cohorts

## Abstract

Chronic obstructive pulmonary disease (COPD) is a prevalent respiratory condition with global implications. Accurate and timely diagnosis is critical; however, traditional diagnostic methods (based on spirometry) show limitations, prompting the search for predictive biomarkers and modern diagnostic techniques. This study explored the validation of COPD-related biomarkers (C-reactive protein, procalcitonin, neutrophil elastase, and alpha-1 antitrypsin) in saliva. A diverse cohort, including healthy non-smokers, healthy smokers, and COPD patients of Polish origin, underwent spirometry and marker analysis. The data correlated with clinical factors, revealing noteworthy relations. Firstly, salivary biomarker levels were compared with serum concentrations, demonstrating notable positive or negative correlations, depending on the factor. Further analysis within healthy individuals revealed associations between biomarker levels, spirometry, and clinical characteristics such as age, sex, and BMI. Next, COPD patients exhibited an enhanced concentration of biomarkers compared to healthy groups. Finally, the study introduced a breathing assessment survey, unveiling significant associations between self-perceived breathing and spirometric and tested parameters. Outcomes emphasized the relevance of subjective experiences in COPD research. In conclusion, this research underscored the potential of salivary biomarkers as diagnostic tools for COPD, offering a non-invasive and accessible alternative to traditional methods. The findings paved the way for improved modern diagnostic approaches.

## 1. Introduction

Chronic obstructive pulmonary disease (COPD) is characterized by airflow limitation in the lungs and pulmonary tissue destruction, often stemming from persistent respiratory tract inflammation [1]. While tobacco smoking remains the primary risk factor, numerous other elements, such as exposure to irritants (biomass smoke, air pollution, and chemicals) and genetic factors (gene polymorphisms, epigenetic modifications, and transcription regulation), significantly contribute to COPD, resulting in diverse disease phenotypes [1,2,3,4]. Among the main genetic variations are polymorphisms in molecules, such as alpha-1 antitrypsin (AAT), tumor necrosis factor (TNF-α), matrix metalloproteinases (MMPs), or antioxidant enzyme genes [4]. Additionally, it was presented that epigenetic mechanisms, including DNA methylation, have the potential to mediate COPD development [5].

Globally, COPD stands as a leading cause of morbidity and mortality, imposing a growing economic burden on many countries. In 2019, an estimated 212.3 million prevalent COPD cases were reported, causing 3.3 million deaths and 74.4 million disability-adjusted life years [6]. Population-based studies reveal variations in COPD prevalence, ranging from 4% to 20% in adults over 40, indicating potential underestimation [7]. The rate of undiagnosed COPD remains high in Europe and beyond, reaching levels of 70% to 90%; consequently, it has been calculated that 28 million Europeans have not yet been diagnosed.

In Poland, approximately 2 million citizens suffer from COPD, which cost the economy 4.5 billion EUR in 2022 [8]. Accurate and early diagnosis of COPD is crucial for reducing patient mortality and alleviating the economic strain associated with advanced disease treatment. Early detection facilitates prompt treatment, potentially delaying the onset of respiratory disorders, including lung cancer [4]. The previous studies investigating the prevalence of COPD in the Polish population have revealed intermediate to high levels of occurrence [9]. For instance, an observation conducted in the Malopolska region reported that approximately 22% of 603 subjects suffered from COPD, ranking among the highest prevalence rates observed in Europe [10]. In this context, our study bears considerable significance in the epidemiological assessment of COPD in Poland, given the absence of population-based studies published since 2011. Furthermore, distinguishing itself from previous prevalent studies, our research incorporates biomarker-based evaluations alongside spirometry and respiratory self-assessment questionnaires.

COPD detection relies on the predicted forced expiratory volume in 1 s (FEV1) percentage and FEV1/forced expiratory volume (FVC) ratio (provided that spirometry is performed correctly); however, it considers only one clinical information [11]. Diagnostics can only be performed by specialists using a spirometer, and due to long waiting times, patients often forego professional diagnosis [12]. Moreover, many measurement errors have been reported [13]. In this context, molecular examinations offer new insights into pathogenesis, improving diagnosis and treatments and facilitating the development of quick diagnostic assays.

Saliva is one of the important body fluids that is well-recognized as a pool of biological markers [14]. Whole saliva is composed of 90% water but also includes peptides and proteins, hormones, sugars, lipids, electrolytes, and several other components [15]. Over the past years, experimental techniques such as mass spectrometry (MS), 2D gel electrophoresis (2-DE), liquid chromatography–tandem mass spectrometry (LC-MS/MS), and free-flow electrophoresis (FFE) separation characterized 2290 proteins in whole saliva, which accounted for approximately 27% of the proteins found in blood serum [16,17,18,19]. Saliva is a non-invasive and safe source of biomarkers dedicated to quick and easy diagnosis and prognosis of diseases that potentially substitute the serum-based diagnostic [14].

Four biomarkers—C-reactive protein (CRP), procalcitonin (PCT), neutrophil elastase (NE), and AAT—are closely related to COPD and were already detected in saliva proteome [20,21,22,23,24,25]. CRP (MW: ~120 kDa) is a member of the pentraxin family, exhibiting diverse roles in both physiological and pathophysiological states. It serves as a valuable biomarker for detecting infection, tissue injury, and inflammation [26]. CRP can be used as a biomarker of COPD [27] and its level is enhanced in COPD patients compared to control groups in previous studies [28]. PCT (MW: ~13 kDa) functions as a prohormone of calcitonin, synthesized by C-cells in the thyroid gland. Intracellular cleavage by proteolytic enzymes results in the production of the active hormone. In healthy individuals, circulating PCT levels are typically below detection limits but significantly increase during episodes of inflammation [29]. Though it may or may not be present in all COPD patients, PCT can give an idea of disease severity [30]. NE protease (MW: ~29.5 kDa) is found in the airways of individuals with pulmonary diseases. When released into the airway milieu in chronic inflammatory airway diseases, NE protease activates inflammation, contributing to the pathogenesis of these conditions [31]. It triggers emphysema by degrading TIMP-1, which is an MMP inhibitor, and NE can trigger reactive oxygen species generating systems by activating factors like IL-8 and TNF-α. Elevated levels of NE have been associated with COPD severity and airway inflammation [32]. AAT deficiency is an inherited disorder characterized by reduced serum levels of alpha-1 antitrypsin (~52 kDa plasma glycoprotein). This deficiency disrupts the protease–antiprotease balance, leading to the destruction of lung structures. Consequently, individuals with a genetic predisposition to alpha-1 antitrypsin deficiency face an elevated risk of developing COPD [33].

The primary objective of this study was to evaluate the efficacy of salivary biomarkers (CRP, PCT, NE, and AAT) in diagnosing COPD in healthy non-smokers, smokers, and COPD patients of Polish origin, compared to traditional spirometry. We then validated the expression of key biomarkers in saliva using the Human Salivary Proteome Wiki and an internal investigation involving specimens from 70 healthy individuals. All values were cross-referenced against serum protein concentrations. Subsequently, we analyzed spirometry parameters and marker concentrations in three distinct groups: healthy non-smokers (n = 70), healthy smokers (n = 70), and COPD patients (n = 140, including both smokers and non-smokers) of Polish origin. These analyses correlated with various clinical factors such as age, gender, BMI, pulmonary infections, allergy history, COVID-19, tobacco addiction, COPD severity, and co-existence of asthma. Additionally, a thorough breathing assessment was conducted and correlated with biomarkers level, spirometry parameters and COPD grades. The results underscored the importance of subjective experiences in the realm of COPD research.

## 2. Materials and Methods

### 2.1. Sample Collection and Population Characterization

From March 2022 to March 2023, our study recruited participants at the Medical University in Warsaw, Poland. The research involved collecting samples from distinct groups: a healthy non-smokers group consisting of 70 individuals (both blood serum and saliva samples were obtained), and a group of healthy smokers comprising 70 individuals from whom saliva samples were collected. Additionally, from March to October 2023, we collected 140 saliva samples from individuals diagnosed with COPD, encompassing both smokers and non-smokers. This COPD group was further categorized based on the severity grades (I–IV) of the disease (Table 1).

The group sizes were determined based on project requirements and case-control study guidelines, as well as insights from prior research. Tobacco smoking was considered a risk factor, and group sizes were calculated using a sample size calculator [34] with a significance level of 0.05 and a statistical power of 80%. The anticipated incidence of smoking was set at 50% in health and COPD populations. For robustness, we chose n = 70 per group in healthy smokers, healthy non-smokers, COPD smokers, and COPD non-smokers.

In adherence to the GOLD (Global Initiative for Chronic Obstructive Lung Disease) standards, spirometry measurements were conducted for all participants. The research strictly adhered to ethical guidelines approved by the Ethical Committee of the Medical University of Warsaw, Poland (no. KB/37/2022). The study was conducted following the Declaration of Helsinki, and the protocol was approved by the Ethics Committee at the Medical University in Warsaw, Poland.

An exhaustive medical interview was administered to all individuals using an electronic case report form (eCRF). This comprehensive form encompassed a range of pertinent details, including gender, BMI, COVID-19 history, vaccination status, COPD severity and prognosis, symptoms like cough, sputum, and chest pain, as well as information about allergies, depression, fatigue during physical exertion, and current medications. A detailed overview of the population’s characteristics is outlined in Table 2.

### 2.2. Saliva and Serum Blood Collection

On the day preceding the collection of materials, participants refrained from consuming over-the-counter medications or dietary supplements. Two hours before sample collection, participants abstained from eating, drinking, smoking, or brushing their teeth. Uniformity was maintained by collecting all samples between 9 and 11 a.m. Additionally, blood samples for testing were obtained at least 2 h post-meal.

The saliva collection process involved participants rinsing their mouths with 10 mL of deionized water. They then tilted their heads forward, allowing saliva to accumulate before gently drooling into a sterile tube (Eppendorf, Hamburg, Germany) until a minimum of 2 mL was attained. Subsequently, collected saliva samples were divided into 0.5 mL portions, promptly placed on ice, and stored at −80 °C until the time of analysis. Before analysis, the thawed saliva underwent centrifugation at 3000 revolutions/minute (rpm) for 15 min.

Peripheral blood, collected in tubes devoid of supplements and ethylenediaminetetraacetic acid vacutainer tubes (BD Bioscience, Franklin Lakes, NJ, USA), underwent centrifugation at 2000 rpm for 15 min. The obtained serum was divided into five aliquots and promptly frozen at −20 °C until subjected to analysis.

### 2.3. ELISA

Biomarker levels were quantified using sandwich ELISA with specific kits: (i) Human C-Reactive Protein ELISA Kit #KHA0031, (ii) PMN (Neutrophil; NE) Elastase, # BMS269, (iii) Human ELISA Kit Procalcitonin (PCT), EHPCT, and (iv) Human ELISA Kit Serpin A1 (AAT), EHSERPINA1. All assays were provided by Invitrogen, Carlsbad, CA, USA, and were conducted following the manufacturers’ instructions.

ELISA measurements were performed in a final volume of 100 μL. CRP detection utilized 3000-fold diluted serum and 3-fold diluted saliva, NE detection employed 100-fold diluted serum and saliva, PCT detection used 2-fold diluted serum and saliva, and AAT detection utilized 3-fold diluted serum/saliva. Samples were added to a pre-coated 96-well microtiter plate specific to CRP, NE, PCT, or AAT. Incubation periods varied: 1 h at 37 °C for CRP assay, 1 h at room temperature for NE assay, and overnight at 4 °C for PCT and AAT assays. After four washes, samples were incubated for 2 h with horseradish peroxidase (HRP)-conjugated anti-CRP/NE/PCT or AAT antibodies. Following four additional washes, substrate solution was added, and plates were incubated for 30 min. The reaction was halted by adding 50 μL of stop solution to each well, resulting in a color change from blue to yellow.

The optical density of each well was measured at 450 nm using a microplate reader (Ledetect96 Microplate Reader, Labexim, Austria). The standard curve provided in the kit was employed to determine biomarker amounts in unknown samples. To calculate the final concentration of biomarkers in serum and saliva, the concentration derived from the standard curve was multiplied by the dilution factor.

### 2.4. Statistical Analysis

Statistical analysis was conducted using GraphPad Prism 6.00 for Windows and GraphPad software based in San Diego, CA, USA. To compare expression levels of CRP, NE, PCT, and AAT among independent groups and their associations with patients’ characteristics (such as age and sex) as well as clinical features of COPD (grade and precisions), the ANOVA Kruskal–Wallis, Student’s *t*-test, or U Mann–Whitney test were employed.

For assessing the relationship between biomarker expression in plasma and saliva, Spearman’s rank correlation was applied. The results of relative expression analysis are presented as mean ± SD for normal distribution. A *p*-value < 0.05 was considered statistically significant with * *p* < 0.05, ** *p* < 0.01, *** p < 0.001, **** *p* < 0.0001.

## 3. Results

### 3.1. Biomarkers (C-Reactive Protein (CRP), Procalcitonin (PCT), Neutrophil Elastase (NE) and Alpha-1 Antitrypsin (AAT)) Presence in Saliva and Serum

**Figure 1:**
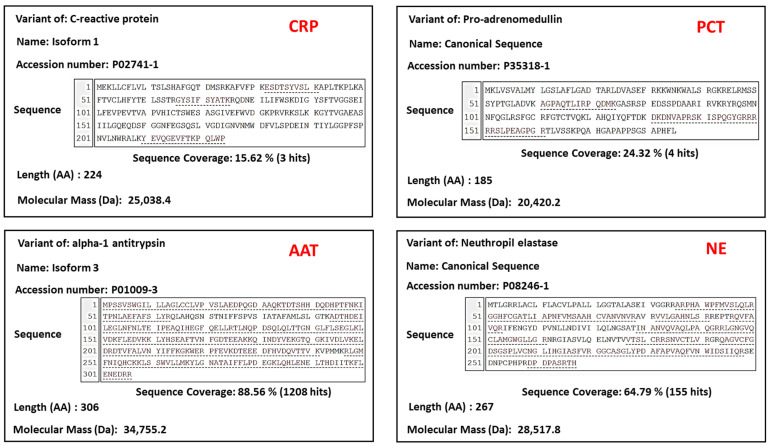
Verification of salivary CRP, PCT, AAT, and NE expression using web portal of human saliva proteins from the Human Salivary Proteome Wiki.

Firstly, all biomarkers (CRP, NE, PCT and AAT presence) were positively verified using the Human Salivary Proteome Wiki [35], where salivary proteins were identified by high-throughput proteomic technologies (Figure 1).

**Figure 2:**
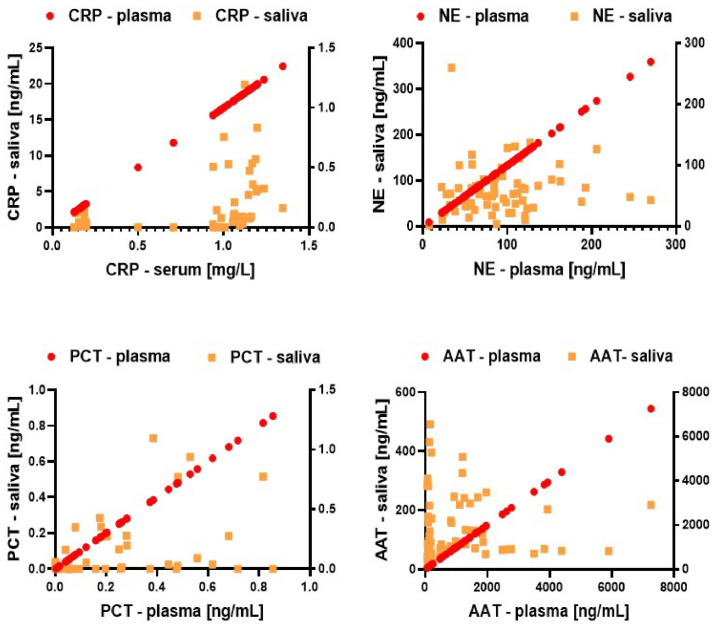
Pearson correlation coefficients between serum and saliva biomarkers sampled from healthy non-smoking study participants.

All expression levels of selected biomarkers were examined using ELISA assays in saliva and blood serum samples collected from 70 healthy volunteers (Figure 2). To explore the relationships between the biomarker levels in blood and saliva, correlation analysis using the Pearson correlation coefficient was conducted across the data sets. The correlation coefficient (r) ranges from +1, indicating a perfect positive correlation, to −1, reflecting a perfect negative correlation.

Commercial ELISA kits were used to quantify both serum and saliva samples, ensuring accurate quantification of concentrations. This allowed investigation of the extent of the correlation of salivary and serum concentrations for 70 samples and the correlation of 4 biomarkers in both types of biological fluids. The average Pearson correlation coefficient (r) for paired biomarkers, such as CRP in serum versus CRP in saliva, demonstrated a positive and statistically significant association for CRP (r = 0.5) and PCT (r = 0.46) (**** *p* < 0.0001). In contrast, NE showed a generally positive correlation (r = 0.08, *p* = 0.46), while AAT displayed a predominantly negative correlation (r = −0.02, *p* = 0.85).

### 3.2. Characterization of Spirometry and Molecular Parameters of Healthy Non-Smoking and Smoking Groups

**Figure 3:**
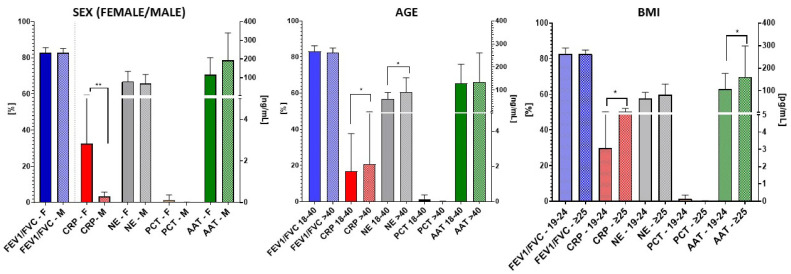
The relationships between the FEV1/FVC ratio, and the salivary biomarkers CRP, NE, PCT, and AAT, within different subgroups (of healthy non-smokers) categorized by sex, age, and body mass index (BMI; calculated by dividing weight in kilograms by the square of the height in meters). The World Health Organization (WHO) defines the following BMI norms: Underweight: BMI less than 18.5; Normal Weight: 18.5–24.9; Overweight: 25–29.9; Obesity > 30. Data are expressed as mean (±S.D.); * = *p* < 0.05, ** = *p* < 0.01.

The key spirometric factor examined was the FEV1/FVC ratio, which represents the proportion of air exhaled in one second relative to the total amount exhaled. This examination was conducted in conjunction with the concentration of biomolecular factors in saliva within the cohort of healthy non-smokers (Figure 3).

Regarding CRP in all three groups, significant differences were observed, particularly with elevated levels in women compared to men (** *p* = 0.003). CRP also displayed an increase in older subjects (>40 years old) (* *p* = 0.01) and individuals with an obesity BMI ≥ 25 (* *p* = 0.03) compared to those categorized as normal weight (BMI = 19–24).

Furthermore, NE exhibited significantly higher levels in the >40-year-old group compared to the younger 18–40 (* *p* = 0.01), whereas AAT was enhanced in individuals with BMI ≥ 25 age group (* *p* = 0.01). However, the remaining differences in biomarker levels within the analyzed group did not reveal significant distinctions.

**Figure 4:**
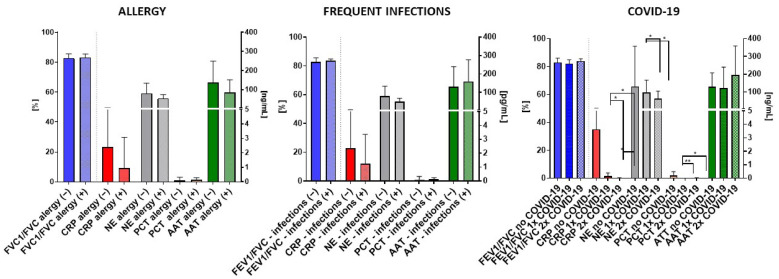
Comparison of FEV1/FVC, and salivary CRP, NE, PCT, and AAT levels within distinct clinical subgroups: with allergy (“−”) vs. without allergy (“+”), individuals with frequent (“infections +”) vs. non-frequent respiratory tract infections (“infections –”), and the frequency presence of COVID-19 (no COVID-19, 1–2x COVID-19 occurrence) among healthy non-smoking individuals. Data are expressed as mean (±S.D.); * = *p* < 0.05, ** = *p* < 0.01.

Subsequently, an evaluation of allergies (including persons with allergies to pollen, cosmetics, insect bites, and food), recurrent respiratory tract infections, and the frequency of coronavirus disease (COVID-19) occurrences was conducted, as depicted in Figure 4.

Remarkably, discernible distinctions were observed exclusively in cases of SARS-CoV-2 infections. Individuals with a singular documented instance of the infection exhibited significantly elevated levels of salivary CRP, NE, and PCT compared to those who experienced the infection 2–3 times (* *p* > 0.05, ** *p* = 0.001). These findings underscore the potential impact of COVID-19 frequency on specific biomarker levels, suggesting a noteworthy association between the frequency/severity of infection and the corresponding salivary biomarker responses.

**Figure 5:**
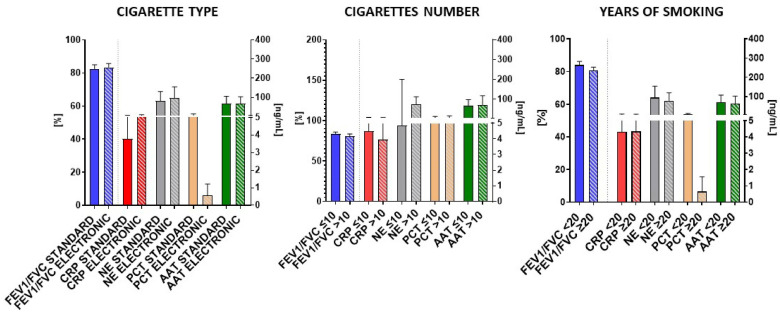
Comparison of FEV1/FVC, and salivary CRP, NE, PCT, and AAT levels across diverse subgroups of smokers based on cigarette type (standard and electronic cigarettes), daily cigarette consumption (≤10 or >10 cigarettes/day), and duration of addiction (<20 or ≥20 years of addiction).

Surprisingly, the group of smokers did not exhibit any notable distinctions in spirometric and molecular parameters concerning the type of cigarettes (standard and electronic), the quantity of cigarettes consumed, or the duration of smoking habits (Figure 5).

### 3.3. Examining Spirometry Parameters and Biomarker Levels within the Groups of Non-Smokers and Smokers with COPD (Comprising Both Non-Smokers and Smokers)

**Figure 6:**
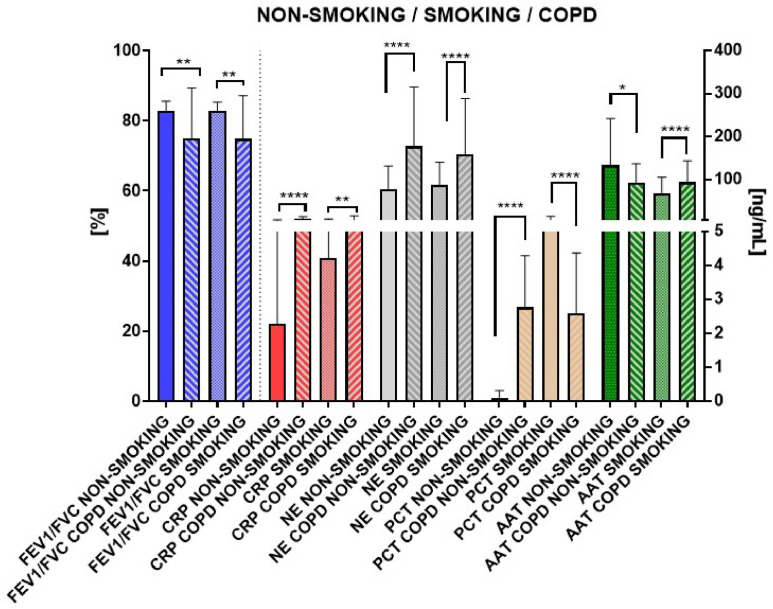
Comparative analysis of spirometry parameters and biomarker levels in healthy and COPD cohorts: non-smoking vs. smoking groups. Data are expressed as mean (±S.D.); * = *p* < 0.05, ** = *p* < 0.01, **** = *p* < 0.0001.

In the final analysis, all groups were systematically compared: healthy non-smoking vs. non-smoking COPD, and healthy smoking vs. COPD smoking (Figure 6).

As a result, all parameters exhibited significant differences. The FEV1/FVC ratio was decreased in both COPD non-smoking and smoking patients compared to their respective counterparts, with a significance of ** *p* = 0.003.

Moving forward, CRP, NE, and PCT levels were notably (**** *p* < 0.0001) increased in non-smoking COPD patients compared to the non-smoking cohort, while AAT demonstrated a decreased pattern (* *p* < 0.5). In smoking COPD patients, CRP (** *p* = 0.047), NE (**** *p* < 0.0001), and AAT (**** *p* < 0.0001) showed higher levels than those addicted to tobacco with a general good condition. Conversely, PCT levels were much lower in COPD saliva compared to smokers. However, PCT levels in non-smoking and smoking COPD groups were on a similar level.

**Figure 7:**
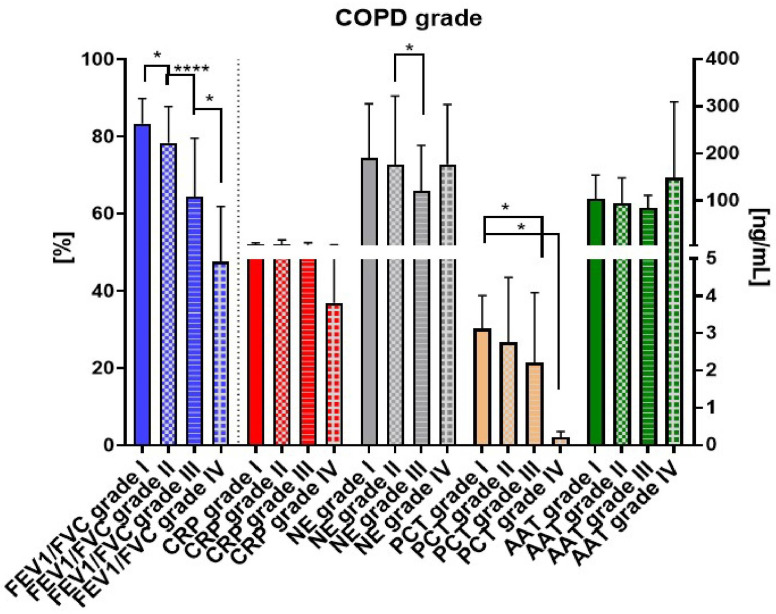
Progressive trends in spirometry and biomarkers across COPD grades: insights from FEV1/FVC ratios and inflammatory markers. Data are expressed as mean (±S.D.); * = *p* < 0.05, **** = *p* < 0.0001.

All assessment parameters consistently exhibited similar patterns, presenting lower levels in more advanced grades of COPD. The FEV1/FVC ratios across grades I–IV showed significant differences in pairwise comparisons: I vs. II (* *p* = 0.02), II vs. III (*** *p* < 0.0001), and III vs. IV (* *p* = 0.01) (Figure 7).

CRP followed a similar trend with decreasing concentrations in subsequent grades; however, the differences were not notable. NE and AAT displayed lower levels in grade II compared to I and lower levels in grade III compared to II, but higher levels in grade IV compared to III. PCT exhibited lower levels in subsequent grade subgroups, with a significant (* *p* = 0.04) difference observed in grades III and IV compared to the grade I group.

**Figure 8:**
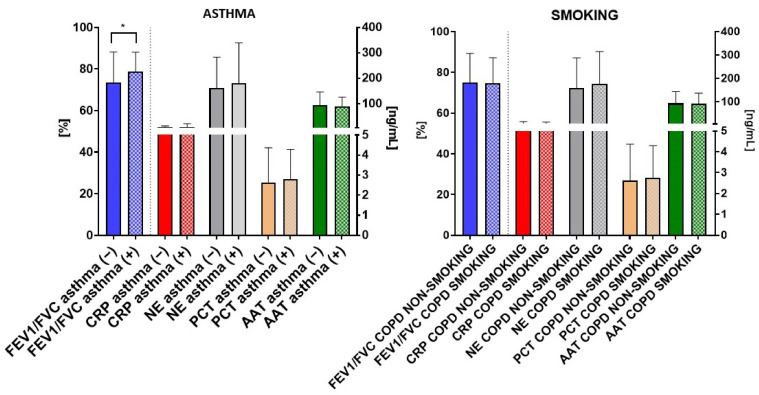
Comparative analysis of respiratory and molecular parameters in COPD patients with (“asthma +”) and without asthma (“asthma –”) and COPD-smoking and non-smoking individuals. Data are expressed as mean (±S.D.); * = *p* < 0.05.

Finally, we conducted a comparison between individuals with and without asthma as a co-existing disease, revealing that patients with asthma exhibited a higher FEV1/FVC ratio compared to COPD patients without asthma (* *p* = 0.04; Figure 8). However, no significant differences were observed in the remaining parameters. Additionally, grouping COPD patients based on smoking and non-smoking status did not reveal any significant differences within the COPD group at a molecular level.

### 3.4. Breathing Assessment of COPD Patients

Breathing self-assessment holds crucial importance in research, particularly within the fields of respiratory medicine and pulmonology. The breathing assessment in this study involved a survey where participants rated their breathing through three questions. In the first question, participants rated their current breath on a scale ranging from “Perfect and very good” to “Bad and very bad”.

The second question assessed how breathing quality affects everyday life: “How does your breathing affect everyday activities, e.g., walking, cleaning, cooking?” with answers on a scale ranging from “Has no effect” to “Strong and very strong”.

The third question concerned the severity of breathing problems during physical activity (less and more advanced): “How do you rate the degree of apnea associated with your activity?” on a scale ranging from “Shortness of breath during intense physical exercise or running”, to “Breathing problems are too severe to leave the house”.

The table below (Table 3) presents the number of cases for each rating category and corresponding values for the FEV1/FVC ratio, and concentrations of CRP, NE, PCT, and AAT.

**Figure 9:**
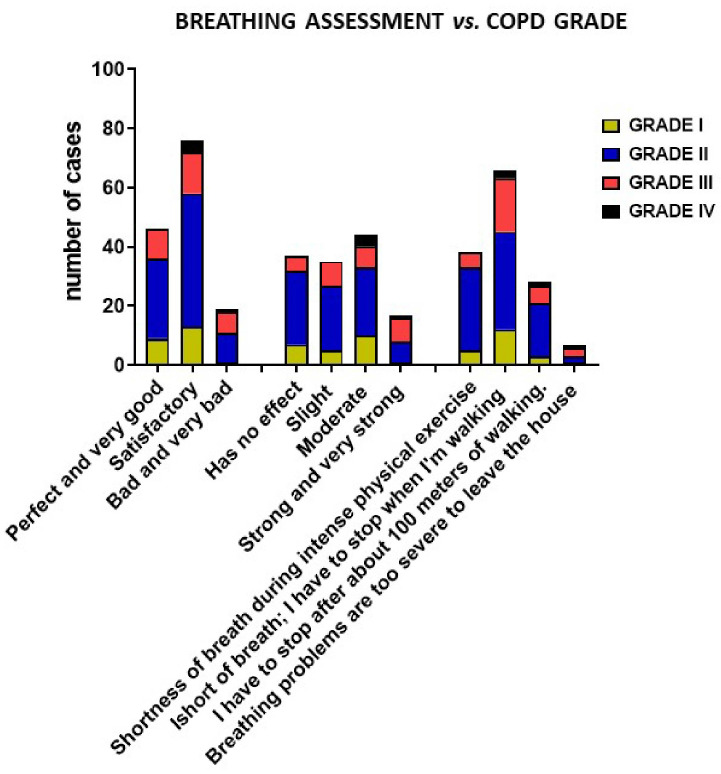
Distribution of COPD patient subgroups into grades I–IV based on breathing assessment questionnaire.

The distribution of the severity of the disease in the given subgroups is presented in Figure 9.

Participants who rated their breathing as “Perfect and very good” (category (1)) had a higher average FEV1/FVC value and biomarker concentrations. In contrast, those who rated their breathing as “Satisfactory” (category (2)) had slightly lower values. Participants rated their breathing as “Bad and very bad” (category (3)) and exhibited the lowest average FEV1/FVC and biomarker concentrations among the categories. The group with the least favorable response exhibited a significantly (* *p* = 0.03) lower level of PCT compared to those with a perfect rating.

Further statistical analyses and correlation assessments provided deeper insights into the relationships between self-rated breathing and this study’s physiological and molecular parameters. The participants were asked to rate how their breathing affects everyday activities such as walking, cleaning, and cooking. As a result, significant differences were observed in various categories, including FEV1/FVC, CRP, NE, PCT, and AAT; the participants who reported “Has no effect” (Category 1) had significantly higher FEV1/FVC values compared to those reporting “Moderate” effect (Category 3) and “Strong and very strong” effect (Category 4) (* *p* = 0.01, *** *p* = 0.0006). Finally, the participants were asked to rate the degree of apnea associated with their activities.

The results showed that participants who reported severe breathing problems that were too severe to leave the house (Category 4) had significantly (**** *p* = 0.0003) lower FEV1/FVC values compared to those experiencing shortness of breath during intense physical exercise or running (Category 1).

## 4. Discussion

COPD poses a significant global health challenge, with diverse contributing factors such as tobacco smoking, environmental exposures, and genetic influences. The prevalence of COPD is substantial worldwide, contributing to substantial morbidity, mortality, and economic burden. Despite being a leading cause of death, COPD remains underdiagnosed, so accurate and timely detection is crucial for effective patient management.

Our study focused on validating salivary biomarkers alongside clinical data and breathing assessments to gauge COPD severity in a Polish cohort. This research is essential given the varied global trends in COPD, particularly pertinent in Poland due to unique challenges like reliance on hard coal for energy [36,37].

Saliva offers a non-invasive alternative for biomarker detection, and our study selected four key biomarkers (CRP, NE, PCT, and AAT) associated with COPD. These biomarkers provide insights into inflammation, infection, and genetic predispositions related to COPD pathogenesis.

The initial step, the verification of biomarkers’ presence within the Human Salivary Proteome Wiki underscored the robustness of biomarker selection. The database use of high-throughput proteomic technologies ensures a systematic and comprehensive identification of proteins present in saliva.

The internal experiments based on cohorts included a healthy non-smokers group (blood serum and saliva samples collected from 70 individuals), a healthy smoker group (with saliva samples collected from 70 individuals) and a group of individuals diagnosed with COPD (consisting of 140 saliva samples from non-smoking and smoking patients). The COPD group was further categorized based on disease severity grades (I–IV) in alignment with the GOLD standards. Notably, the distribution of clinical characteristics varied among the groups providing a comprehensive snapshot of the study population.

The utilization of commercial ELISA kits with standards for precise quantification enabled the thorough investigation of correlations between salivary and serum concentrations across 70 samples obtained from healthy individuals. The average Pearson correlation coefficient (r) revealed significant associations for paired biomarkers in both biological fluids. According to the results, CRP and PCT exhibited a positive and statistically significant correlation between serum and saliva concentrations (r = 0.5 and r = 0.46, respectively, **** *p* < 0.0001), NE demonstrated a generally positive correlation (r = 0.08, *p* = 0.46), whereas AAT displayed a predominantly negative correlation (r = −0.02, *p* = 0.85). These findings underscore the potential utility of salivary biomarkers as reflective indicators of systemic biomarker concentrations, providing valuable insights into their interplay and offering a non-invasive way for biomarker assessments; however, their presence and quantity should be verified individually [19,38].

The investigation into spirometry and molecular parameters among healthy non-smokers, smokers, and COPD patients revealed associations that provide valuable insights into physiology. Significant variations in CRP levels across groups were observed, particularly with higher concentrations in women (*** *p* = 0.0003), older subjects (>40 years old; ** *p* = 0.001; accompanied by higher NE concentration * *p* = 0.01) and individuals with an overweight body mass index (BMI ≥ 25; * *p* = 0.03; accompanied by AAT concentration * *p* = 0.01). Sex differences in C-reactive protein (CRP) levels intensify notably after the age of 15, influenced in part by the inflammatory challenges accompanying the physiological and behavioral changes of adolescence, with a more pronounced impact on females [39]. It should be underlined that the gender ratio varied among the three groups under investigation, with a predominance of women in each group. This discrepancy suggests that women are more inclined to undergo regular medical visits (in the case of the healthy groups) and seek medical attention for respiratory symptoms (in the case of the COPD group) compared to men.

CRP levels exhibited a discernible trend within a defined cohort, encompassing volunteers aged 20 to 70 years, organized into distinct groups with a 10-year interval between them [40]. With aging, healthy human donors have elevated NE levels in the airways; the percentage and number of NEs in bronchoalveolar lavage fluid are significantly higher than those in young controls [41,42]. Furthermore, individuals classified as overweight (BMI = 25–29.9 kg/m^2^) or obese (BMI ≥ 30 kg/m^2^) demonstrated an elevated CRP and AAT level compared to their normal-weight counterparts (BMI < 25 kg/m^2^). This association aligns with the characteristic features of metabolic syndrome, marked by the release of inflammatory adipokines from adipose tissue [43,44].

Interestingly, analyses of COVID-19 infections revealed distinctive patterns, especially in cases of SARS-CoV-2 infections. Subjects with no documented COVID-19 instance exhibited significantly elevated levels of CRP, NE, and PCT compared to those with 1 and 2 occurrences (** *p* = 0.001), suggesting a potential link between infection frequency/severity and salivary biomarker responses [45,46,47]. Serum CRP, NE, and PCT were associated with COVID-19 infection, mortality, and predictions of disease; however, data did not connect the frequency of infection.

No significant differences were observed in spirometric and molecular parameters among smokers based on cigarette type, quantity, or duration of smoking habits.

In our study, COPD non-smoking and smoking patients exhibited significantly lower FEV1/FVC and significant differences in CRP, NE, PCT, and AAT levels in saliva compared to healthy non-smoking and smoking individuals, respectively. CRP, NE, and PCT levels were notably (**** *p* < 0.0001) increased in non-smoking COPD patients compared to the non-smoking cohort, while AAT demonstrated a decreased pattern (* *p* < 0.5). In smoking COPD patients, CRP (** *p* = 0.047), NE (**** *p* < 0.0001), and AAT (**** *p* < 0.0001) showed higher levels than those addicted to tobacco with a general good condition. Conversely, PCT levels were much lower in saliva from the COPD patients compared to smokers; however, PCT levels in non-smoking and smoking COPD groups were on a similar level.

Patel et al. analyzed salivary CRP and observed a significant increase in CRP levels in COPD compared to NS, but not to smokers [48]. In our study, healthy individuals showed lower saliva CRP values, which were almost double in smokers and more than triple in COPD patients. As previously described, smoking also leads to high CRP values both in serum and saliva as seen in previous studies coinciding with our results. In an experiment for checking COPD exacerbation in hospitalized patients, the mean CRP values were higher at admission than during discharge or follow-up [49]. CRP levels were found to be elevated among the smokers than the non-smokers. A similar study showed a decrease in serum CRP levels based on a week of hospitalization in COPD patients [50].

In a study of 30 COPD subjects, the average serum NE level for stable patients was 2454 ng/mL (1460–4125 ng/mL). This study also proved that NE was a valuable biomarker for predicting COPD exacerbations, especially due to bacterial infections [51]. In healthy and I, II, III, and IV-stage groups, the NE level showed an increasing trend for the more advanced COPD grade [52].

Notably, Patel et al. conducted a study revealing elevated NE levels in saliva samples from smokers compared to non-smokers and even COPD patients, with an increase in exacerbated patients [48]. Furthermore, the study highlighted a decrease of nearly 60 ng/mL in NE levels for every 10-year increment in age, irrespective of COPD treatment type. However, this investigation failed to establish a correlation between NE levels and the severity of COPD. In our study, the NE levels for COPD patients were higher than the smokers and non-smokers. The NE concentration was significantly decreased in the III-grade group compared to the I-grade cohort and within I, II, and IV-grade groups were on a similar level. The intricate interplay between NE levels, smoking status, age, and COPD grades underline the dynamic nature of this biomarker in the context of respiratory health.

In healthy individuals, the levels of PCT are below 0.01 g/L but can increase up to 100 g/L during severe infections and had a half-life of 25–30 h in blood serum [53,54]. In a study analyzing the relation of PCT with acute exacerbation of COPD, the control group of healthy individuals showed lower PCT levels in serum than the stable and exacerbated COPD patients [55]. The salivary PCT levels were found to be higher in the smoker group compared to the non-smokers and COPD patients. The study also showed that PCT level correlations from the saliva samples are quite comparable to serum PCT levels [48]. In our study similarly, the COPD patients showed an average PCT level of 2.7(±1.7) ng/mL from saliva samples while the healthy smokers and non-smokers showed 5.9 ng/mL and 0.09 (±0.2) ng/mL, respectively. It would be interesting to elucidate the cause of higher PCT values in smokers even more than the COPD patients. It is possible that smoking itself triggers inflammation within the body leading to high PCT values which may be masked due to other responses during COPD progression.

In COPD, the AAT-NE imbalance leads to emphysema in the lower airway. The circulating AAT threshold falls below 50 mg/dL from a normally abundant 100–200 mg/dL [56]. This also adds up to the problems by recruiting additional neutrophils to the airways for remodeling causing complications in COPD which are aggravated by smoking due to the presence of cotinine (metabolized form of nicotine) acting as an oxidizing agent [57,58]. In a study based on AAT and cotinine levels in smokers and non-smokers, AAT was significantly decreased while cotinine levels increased [59]. The AAT levels from saliva in our study also showed a significant decrease in AAT levels from non-smokers to smokers. In COPD patients, however, the AAT levels were higher than in smokers. In another study, serum AAT levels were found to rise with COPD severity [43]. This study also reported a similar drop in CRP levels in Stage IV of COPD compared to the other 3 stages. This might indicate a difference in the patient’s physiology as the disease reaches its final stage, which needs further investigation.

According to the breathing assessment, we determined the level of association between the internal feeling of individuals of current breath and disability due to breathlessness (including the MRC dyspnea scale), and other variables are used to measure the severity and impact of COPD. It, therefore, appears that the correlates of disability due to breathlessness may vary with the level of disability and correlate with spirometry and molecular patterns. It was already shown that the MRC dyspnea scale proves to be a straightforward and reliable method for categorizing COPD patients based on their disability, offering a valuable complement to FEV1 in the comprehensive classification of COPD severity; also in the remote monitoring [60,61]. In conclusion, this study has shown that subjective assessment can provide a simple and valid method of categorizing patients in terms of their disability due to COPD. Altogether, a complete analysis including spirometry, biomarker and breathing analysis, and comprehensive patient profiling can provide a detailed and reliable diagnosis for COPD severity.

To summarize, the assessment of saliva biomarkers in connection with breathing assessment offers valuable insights into diagnosing COPD and its exacerbations. By monitoring fluctuations in these biomarkers against clinical thresholds, we can develop rapid, reliable, and non-invasive diagnostic tools. A significant challenge in COPD diagnosis, particularly in Western countries, is patients’ inability to recognize respiratory issues, coupled with limited access to spirometry for outpatients [62]. In this context, rapid diagnosis based on salivary biomarkers not only aids in prognostication but also facilitates timely and accurate treatment and disease management [63].

## 5. Conclusions

In this work, we investigated how salivary biomarkers (CRP, NE, PCT, and AAT) correlate with lifestyle, clinical data, and breathing assessments. Our results provide insight into the crucial factors required for diagnosing, monitoring, and developing targeted therapeutic strategies for COPD and related respiratory conditions. The dynamic interplay between the different factors provides valuable insights into the underlying mechanisms of inflammation, tissue damage, and exacerbations in the context of respiratory diseases.

In summary, our study contributes to the growing body of knowledge on COPD by validating salivary biomarkers and exploring their correlations with clinical parameters for the first time in a Polish population study. The findings emphasize the potential of non-invasive diagnostic methods and subjective assessments for effective COPD management. All information was a basis for our new modern diagnostic tool, biosensor with application, which was developed during the executed project.

## Figures and Tables

**Table 1:** Inclusion and exclusion criteria for healthy non-smokers, healthy smokers, and COPD patients.

No.	Inclusion Criteria for Non-Smokers and Smokers:	Exclusion Criteria for Groups
**1**	aged > 18 years (male or female)	for healthy smoking and non-smoking cohorts: individuals diagnosed with COPD or other respiratory diseases
**2**	general good health condition	pregnancy or breastfeeding
**3**	(1) for the non-smoking group: the non-smoking individuals; (2) for smoker’s cohort: addicted to nicotine	the likelihood of non-cooperation or participation in another clinical trial, a history of addiction to nicotine (for the non-smoking group), drugs, or alcohol, an implanted bioelectric device, such as a pacemaker, hearing aid, orthodontic appliance, or metal wires in the mouth
**No.**	**Inclusion Criteria for COPD Patients:**	
**1**	aged > 18 years (male or female)	
**2**	diagnosis of COPD at least 12 months before sample collections	

**Table 2:** Characteristics of study populations of healthy non-smokers, healthy smokers, and COPD individuals.

Clinical Characteristic	Healthy Non-Smokers n = 70	Healthy Smokers n = 70	COPD n = 140
Gender			
Female	55 (79%)	51 (73%)	73 (52%)
Male	15 (21%)	19 (27%)	67 (48%)
Age			
18–39	30 (43%)	30 (43%)	0 (0%)
40–55	28 (40%)	24 (34%)	4 (3%)
>55	12 (17%)	16 (33%)	136 (97%)
BMI (kg/m^2^)			
18.5–24.9	45 (64%)	30 (43%)	41 (29%)
25.0–29.9	18 (26%)	27 (39%)	54 (39%)
>30	7 (10%)	13 (18%)	45 (32%)
Regular Medication (Unrelated COPD)			
With Medicines (medicine for blood pressure and thyroid medications)	12 (17%)	16 (23%)	109 (79%)
Without Medicines	58 (83%)	54 (77%)	31 (21%)
Comorbidities			
Yes	N/A *	N/A	82 (59%)
No	N/A	N/A	58 (41%)
COVID-19			
0	33 (47%)	35 (50%)	96 (69%)
1	29 (41%)	24 (34%)	44 (31%)
≥2	8 (11%)	11 (16%)	0 (0%)
Doses OF COVID-19 Vaccines			
0	8 (11%)	4 (6%)	12 (9%)
1	0 (0%)	4 (6%)	0 (0%)
2	14 (20%)	16 (23%)	31 (22%)
3	43 (61%)	32 (46%)	58 (41%)
4	5 (8%)	14 (20%)	39 (28%)
Allergy			
Yes (allergy to pollen, cosmetic, insect bites, food)	6 (9%)	11 (16%)	122 (87%)
No	64 (91%)	59 (84%)	18 (13%)
Frequent Respiratory Infections			
Yes	5 (7%)	9 (13%)	13 (9%)
No	65 (93%)	61 (87%)	127 (91%)
COPD Grade			
I	N/A	N/A	23 (16%)
II	N/A	N/A	81 (58%)
III	N/A	N/A	31 (22%)
IV	N/A	N/A	5 (4%)
Years since the COPD Diagnosis			
1–5	N/A	N/A	75 (54%)
6–10	N/A	N/A	36 (26%)
>10	N/A	N/A	29 (20%)
Bronchial Asthma			
Yes	0	0	38 (27%)
No	0	0	102 (33%)
Prediction for Patient			
Very good and good	N/A	N/A	22 (16%)
Moderate good	N/A	N/A	45 (32%)
Moderate bad	N/A	N/A	45 (32%)
Bad and very bad	N/A	N/A	28 (20%)
Tobacco Addiction			
Non-smoking	70 (100%)	N/A	87 (62%)
Standard cigarettes	N/A	45 (64%)	53 (38%)
Electronic cigarettes	N/A	25 (36%)	0 (0%)
Years of Smoking			
0	N/A	0 (0%)	87 (62%)
1–19	N/A	40 (57%)	1 (1%)
≥20–29	N/A	21 (30%)	5 (3%)
≥30	N/A	9 (13%)	47 (34%)
Numbers of Cigarettes/Day			
0	N/A	0 (0%)	87 (62%)
1–9	0	47 (67%)	7 (5%)
≥10	0	23 (33%)	46 (33%)
Exposure to Dust			
No	0 (0%)	0 (0%)	0 (0%)
Yes	70 (100%)	70 (100%)	140 (100%)
Pain in Chest			
No	70 (100%)	66 (94%)	136 (97%)
Yes	0 (0%)	4 (6%)	4 (3%)
Cough without Infection			
No	70 (100%)	70 (100%)	19 (14%)
Yes	0 (0%)	0 (0%)	121 (86%)
Wheezing			
No	70 (100%)	69 (99%)	80 (57%)
Yes	0 (0%)	1 (1%)	60 (43%)
Short of Breath during Physical Activity			
No	70 (100%)	66 (94%)	18 (13%)
Yes	0 (0%)	4 (6%)	122 (87%)
Difficulty Inhaling			
No	70 (100%)	66 (94%)	95 (68%)
Yes	0 (0%)	4 (6%)	45 (32%)
Sputum			
No	70 (100%)	1 (1%)	26 (19%)
Yes	0 (0%)	69 (99%)	114 (81%)
Depression			
No	70 (100%)	70 (100%)	2 (1%)
Yes	0 (0%)	0 (0%)	138 (99%)
Cyanosis			
No	70 (100%)	70 (100%)	140 (100%)
Yes	0 (0%)	0 (0%)	0 (0%)

* N/A—not applicable.

**Table 3:** Breathing assessment questionnaire.

Breathing Assessment (Answer Category)	How Do You Rate Your Breathing?					
	Number of Cases	FEV1/FVC Value	CRP	NE	PCT	AAT
**(1) Perfect**	46	77.8 ± 10	8.4 ± 11.9	170.9 ± 153	2.8 ± 1.4	83.6 ± 30
**(2) Satisfactory**	76	74.0 ± 15	6.5 ± 4.5	174.5 ± 125	2.6 ± 1.8	99.6 ± 57
**(3) Bad and very bad**	18	71.6 ± 13	8.5 ± 4.7	120 ± 84	2.0 ± 1.4	90.5 ± 31
**Significant differences**		-	-	-	(1) vs. (3) * *p* = 0.03	
	**How Does Your Breathing Affect Everyday Activities, e.g., Walking, Cleaning, Cooking?**					
**(1) Has no effect**	39	79.6 ± 9	8.1 ± 6.7	180 ± 115	3.1 ± 1.7	80.3 ± 23
**(2) Slight**	36	76.6 ± 9	6.6 ± 4.4	157.9 ± 137	2.7 ± 1.5	100.9 ± 67
**(3) Moderate**	48	73.7 ± 12	7.3 ± 10	187.8 ± 151	2.6 ± 1.7	98.6 ± 45
**(4) Strong and very strong**	17	64 ± 22	4.5 ± 4.3	92.6 ± 53	1.6 ± 1.2	90.3 ± 40
**Significant differences**		(1) vs. (3) * *p* = 0.01 (1) vs. (6) *** *p* = 0.0006 (2) vs. (4) ** *p* = 0.007 (3) vs. (4) * *p*= 0.03	(1) vs. (4) * *p* = 0.04	**-** (1) vs. (4) ** *p* = 0.004 (3) vs. (4) * *p* = 0.01	**-** (1) vs. (4) ** *p* = 0.001 (2) vs. (3) * *p* = 0.01 (3) vs. (4) * *p* = 0.01	**-** (1) vs. (3) * *p* = 0.02
	**How Do You Rate the Degree of Apnea Associated with Your Activity?**					
**(1) Shortness of breath during intense physical exercise or running**	38	77.7 ± 10	5.8 ± 3.9	169.3 ± 153	2.7 ± 1.9	86.5 ± 24
**(2) I get short of breath faster than people my age when walking. I have to stop when I’m walking at my own pace on a level.**	67	75.3 ± 12	8.7 ± 10	169.2 ± 128	2.8 ± 1.5	92.4 ± 47
**(3) I have to stop after about 100 m of walking.**	28	75.4 ± 11	5.7 ± 4.5	169.5 ± 122	2.3 ± 1.4	108 ± 70
**(4) Breathing problems are too severe to leave the house**	7	54.6 ± 28	2.8 ± 2.4	112 ± 66	1.5 ± 1.2	76 ± 30
**Significant differences**		(1) vs. (4) *** *p* = 0.0003				

## Data Availability

Data are contained within the article.

## Abbreviations

COPD	chronic obstructive pulmonary disease
AAT	alpha-1 antitrypsin
FEV1	forced expiratory volume in 1 s
FVC	forced expiratory volume
TNF	tumor necrosis factor
MMP	matrix metalloproteinase
MP	mass spectrometry
2-DE	two-dimensional gel electrophoresis
LC-MS/MS	liquid chromatography-tandem mass spectrometry
FFE	free-flow electrophoresis
CRP	C-reactive protein
PCT	procalcitonin
NE	neutrophil elastase
GOLD	global initiative for chronic obstructive lung disease
CRF	case report form
BMI	body mass index
WHO	The World Health Organization
